# Rapid quantification of sequence repeats to resolve the size, structure and contents of bacterial genomes

**DOI:** 10.1186/1471-2164-14-537

**Published:** 2013-08-08

**Authors:** David Williams, William L Trimble, Meghan Shilts, Folker Meyer, Howard Ochman

**Affiliations:** 1 Department of Ecology & Evolutionary Biology, Yale University, New Haven, Connecticut 06520, USA; 2 Institute for Genomics & Systems Biology, University of Chicago, 5800 S Ellis Ave, Chicago, Illinois 60637, USA; 3 Mathematics & Computer Science Division, Argonne National Laboratory, 9700 South Cass Avenue, Argonne, Illinois 60439, USA

**Keywords:** *K*-mer, Genome assembly, Repetitive elements, Bacterial evolution

## Abstract

**Background:**

The numerous classes of repeats often impede the assembly of genome sequences from the short reads provided by new sequencing technologies. We demonstrate a simple and rapid means to ascertain the repeat structure and total size of a bacterial or archaeal genome without the need for assembly by directly analyzing the abundances of distinct *k*-mers among reads.

**Results:**

The sensitivity of this procedure to resolve variation within a bacterial species is demonstrated: genome sizes and repeat structure of five environmental strains of *E. coli* from short *Illumina* reads were estimated by this method, and total genome sizes corresponded well with those obtained for the same strains by pulsed-field gel electrophoresis. In addition, this approach was applied to read-sets for completed genomes and shown to be accurate over a wide range of microbial genome sizes.

**Conclusions:**

Application of these procedures, based solely on *k*-mer abundances in short read data sets, allows aspects of genome structure to be resolved that are not apparent from conventional short read assemblies. This knowledge of the repetitive content of genomes provides insights into genome evolution and diversity.

## Background

New sequencing technologies, by generating hundreds of millions of reads from multiplexed samples, allow the rapid and simultaneous acquisition of many genome sequences, and foster comparative analyses of closely related organisms. Despite achieving high coverage, the short reads generated by many sequencing platforms permit only partial assembly of genomes, due largely to the presence of numerous classes of repetitive sequences. Only regions of unique, single copy sequence and repeat regions shorter than the read length can be accurately assembled, yielding draft genomes that consist of unordered contigs separated by gaps of unspecified size [1]. These technological limitations conceal the size and organization of a genome because the number, contents and distribution of repeat arrays remain unknown. To resolve the total size and repeat organization of a genome typically demands a complete genome assembly, which is ultimately obtained by linking contigs through combinatorial strategies and additional sequencing [2], which may sometimes be aided by additional finishing approaches, such as optical mapping [3].

The number, types and configuration of repeated sequences varies greatly within and across species. Even bacterial genomes, which consist mostly of single-copy protein-encoding genes, can contain multiple classes of repetitive sequences that can prevent their assembly and analysis. Bacterial genomes encode as many as 15 ribosomal DNA operons, which can each span several kilobases and are of nearly identical sequence within most organisms, complicating the assembly of their genomes [4]. Even more problematic are the shorter repetitive elements and sequence repeats present in bacterial genomes [5,6]. For example, most bacteria harbor multiple classes of insertion sequence (IS) elements, which average about a kilobase in length and whose copy numbers are highly variable, sometimes reaching hundreds of copies per genome [7,8]. In addition, there are several other types of repetitive sequences, including the 21-to-65-nt repetitive extragenic palindromic (REP) sequences [9], the 127-bp palindromic enterobacterial repetitive intergenic consensus (ERIC) sequences [10], and the bacterial interspersed mosaic elements (BIME) [11], each of which can be dispersed throughout the genome or exist in tandem arrays [12].

Heterogeneity in the numerous classes of repetitive sequences contributes to the genome size variation that has been observed in many bacterial species. For example, isolates of *Escherichia coli* can differ by up to 30% in genome size, with sequenced strains ranging from 4.5 to 5.9 Mb [13-15]. Some of this size diversity is also attributable to the differential accumulation of plasmids and prophages. Accessory elements can also complicate the repeat structure of genomes because the multiple prophages within a genome can encode the same genes, and plasmids are often maintained in very high copy numbers.

Because high-coverage, short-read data contain information about repeat content that is not apparent in draft assemblies, we developed a procedure to estimate the size and repeat content of genomes from raw sequence data. This method relies on the analysis of the frequencies of overlapping fixed-length sequences (*k*-mers) [16], thereby exploiting a computationally efficient and scalable analytical technique that underlies many search and assembly algorithms. To date, *k*-mer-based algorithms have served as the basis for efficient similarity searching [16], vector removal [17], graph-based, short-read assembly [18,19], short-read error correction [20,21], estimation of genome repeat structure [22], comparison of whole genomes [23], identification of anomalous genome regions [24,25] and binning of sequence fragments from different sources [26,27]*.*

We show that direct analysis of the abundances of unique 21-mers (icosihenamers) recovered from sets of whole-genome shotgun short read sequences yields robust estimates of total genome size and of the proportion of the genome represented by repeats of each copy number. Furthermore, sequence coverage can be estimated, sequence quality can be characterized, and sample contamination can often be diagnosed. Therefore, resolution of the repeat architecture of a genome can facilitate comparisons of genome size, structure and complexity, help the assembly of accurate genome sequences, and improve confidence in genome assemblies.

## Results and discussion

### Analysis of short-read datasets from novel *E. coli* strains

Our choice of *k*-mer length, 21, was determined empirically and reflects the minimum size for these genomes that can reliably distinguish small repeats from single-copy sequences. Abundance analysis using smaller *k*-mers is sensitive to shorter repeats, resulting in larger proportions of sequence inferred to occur in multiple copies (Additional file 1: Table S1) and *k*-mers shorter than 15 nucleotides in length are more likely to match by chance alone. The wider range of repeat lengths spanned by longer *k*-mers causes abundance analysis to be less sensitive to short repeats, such that smaller fractions of the genome will be classified as part of a repeat. Discussions of *k*-mer length have treated it as a parameter to be optimized [28,29] and as a sensitivity/specificity tuning parameter for search and assembly [28,30]. It should be noted that a *k*-mer length of 21 is biologically relevant: it is short enough to resolve small repeated elements that occur in *E. coli*, such as REP and ERIC sequences [10,24] but long enough to distinguish between single copy protein-encoding regions by spanning the sequence that defines such regions as unique.

There are several methods to efficiently enumerate *k*-mers, including dense representations [31], hashing [32], and probabilistic data structures [33]. Technical limitations on computer memory and storage space make dense representations unreasonable for *k*-mers longer than *k* = 18; however, the hashing implementation used here (*i.e*., Jellyfish [[32]]) is applicable to *k*-mers as long as 31, and can in principle be applied to very large datasets (10^12^ bp). Due to reverse-complement degeneracy, the total number of possible *k*-mers is approximately (½) 4^*k*^, and for *k* = 15 or greater, there are many more possible *k*-mers (>500 million) than basepairs in the largest observed bacterial and archaeal genomes (<13 Mb). The scarcity of long *k*-mers, together with the fact that most microbial sequence is non-redundant, has the consequence that most *k-*mers that do occur are present only once, contain sequence from single copy genes, and map to unique locations of the genome.

**Figure 1:**
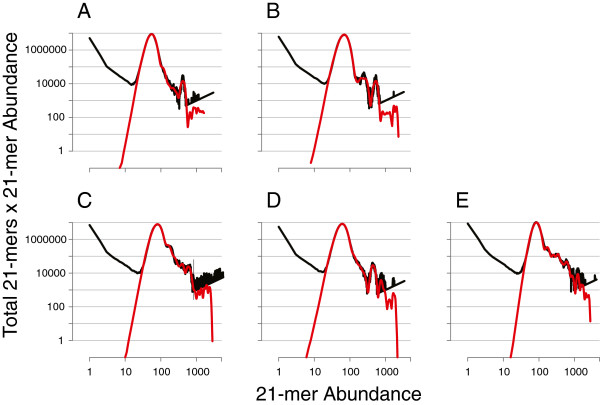
**Abundance histograms of icosihenamers (21-mers) for five strains (A–E) of *E. coli.*** Black lines represent the total number of distinct 21-mers at each abundance value (as present in the *Illumina* short-read dataset for a strain), and red lines are the best-fit model for each of the empirical 21-mer spectra. To increase the area within the plot containing peaks, total numbers of distinct 21-mers are multiplied by their corresponding 21-mer abundances. This transformation does not affect the model fitting, and estimates of repeat structure and genome size remain unaffected. Panel labels are as follows, **A**: strain A_03_34; **B**: strain B_04_28; **C**: strain C_04_22; **D**: strain D_04_27, **E**: strain E_01_37.

The abundance histograms (Figure 1A – E) display the number of distinct 21-mers at each abundance (black line) for the short-read datasets generated for five environmental strains of *E. coli*. To improve visualization of these spectra, we transformed the data by multiplying the total 21-mers at an abundance by that abundance. These histograms share several features denoting similarities in the read sets produced for each genome. Starting in the upper left of each plot are numerous, but low-abundance, 21-mers: these represent the unique and rare 21-mers produced by sequencing errors and are therefore not relevant to assessments of genome size and architecture. Next along the curve is the principal peak comprised of 4–5 million different 21-mers present at approximately the same abundance. These represent 21-nucleotide sequences that are present in a single copy in the underlying genome. The horizontal position of the principal peak, representing the mode of the 21-mer abundance, is proportional to sequencing coverage. For sequencing runs of the *E. coli* strains that we examined, sequence coverage ranged from 55× in strain B to 85× in strain A, resulting from variation in the number of sequencing reads produced for each strain.

The multi-modal distributions of 21-mers at abundances above that of the principal peak reflect primarily the several classes of repetitive sequences that are present at different copy numbers in a genome, although unevenness in read coverage of the target genome can cause distortion of the peak shape. The successive peaks occur at multiples of the principal peak’s abundance, each of which reflects the repeat copy number of the sequence contained in those 21-mers, and the area under each peak is determined by the amount of unique sequence at that copy number. In all five *E. coli* genomes analyzed, the first peak is the largest, consistent with the expectation that complete *E. coli* genome sequences, and bacteria in general, are relatively gene-rich and consist primarily of single copy sequences.

To help interpret the 21-mer spectra of unassembled short-reads, we also examined the relationship between 21*-*mer abundances and the distribution of repeated sequences in a fully assembled genome. The abundance frequencies of 21-mers in the complete *E. coli* DH1 genome (Table 1) reveals a total of 4,494,886 unique 21-mers (representing 97% of the genome), and another 33,614 21-mers present between two and 75 times. It is also notable that 5,132 21-mers in the *E. coli* DH1 genome were repeated seven times, corresponding to sequences common to the seven near-identical ribosomal DNA (*rrn*) operons (which includes the genes encoding the 16S and 23S ribosomal subunits) and to the *insD* IS2 transposase, which is also at seven copies in this genome. Additionally, 1,240 21-mers are repeated more than 10 times, most of which correspond to the *insH* IS5 transposase, present fifteen times in the DH1 genome.

**Table 1:** **Size and repeat structure of *E. coli *genomes estimated by icosihenamer (21-mer) analysis**

	**Genome sequence (bp)**											
	**Strain A_03_34**		**Strain B_04_28**		**Strain C_04_22**		**Strain D_04_27**		**Strain E_01_37**		**DH1 (reference)**	
**Copy number**^**a**^	**Unique**	**Total**	**Unique**	**Total**	**Unique**	**Total**	**Unique**	**Total**	**Unique**	**Total**	**Unique**	**Total**
1×	4,650,095	4,650,095	4,834,774	4,834,774	4,590,007	4,590,007	5,002,844	5,002,844	4,836,194	4,836,194	4,494,886	4,494,886
2×	34,059	68,119	5,364	10,728	177,520	355,041	45,770	91,541	111,882	223,764	14,578	29,196
3×	3,550	10,649	8,511	25,532	25,052	75,156	9,630	28,890	34,198	102,595	6,959	20,877
4×	1,158	4,632	6,296	25,183	8,270	33,079	2,235	8,939	30,549	122,197	2,072	8,288
5×	845	4,223	24	119	5,855	29,277	447	2,236	8,777	43,887	1,874	9,370
6×	0	0	286	1,714	2,271	13,627	196	1,175	4,611	27,665	1,415	8,490
7×	2,208	15,455	1,283	8,982	2,786	19,505	5,489	38,420	8,167	57,168	5,132	35,924
8×	2,566	20,530	3,311	26,486	4,028	32,222	3,170	25,360	3,301	26,405	213	1,704
9×	0	0	41	365	1	13	99	887	662	5,961	23	207
10×	0	0	6	64	424	4,242	6	56	989	9,888	26	260
11 × -20×	107	1669	24	329	679	10,796	1,331	17,155	3,080	45,197	1,240	18,757
21 × -79×	14	333	32	835	709	17,629	70	1,884	68	1,590	62	2,728
Cumulative totals:		4,775,705		4,935,111		5,180,594		5,219,387		5,502,511		4,630,687

^a^Each row corresponds to the number of nucleotides and the total amount of genome sequence inferred from the mixed Poisson model fit to each peak of the 21-mer spectrum of short reads for each of the novel *E. coli* strains (Figure 1A-E), and from direct counts of 21-mer in the *E. coli* DH1 genome sequence.

### Relating abundance histograms to genome repeat structures

The number of 21-mers that reside under each peak in an abundance histogram corresponds closely to the number of basepairs of sequence at a particular repeat copy-number. To estimate the amount of unique sequence under each peak and to gain insight into the repeat structure of each target genome, we applied a maximum likelihood estimator to model the distribution of 21-mers in each histogram spectrum (red lines in Figure 1). This approach is similar to but has advantages over the method used by Li & Waterman [22] to estimate repeat structure from sequence reads. In our implementation, the inference of sequence repeats is guided by the natural property of 21-mer abundances to occur at integer multiples of the modal *k*-mer abundance (the principal peaks in Figure 1). Whereas our fitting procedure exploits this property to stabilize the likelihood optimization, the implementation of Li & Waterman [22] uses the expectation-maximization algorithm. In addition, our approach was evaluated using high-coverage, short-read raw sequencing data and is optimized for current technologies.

The amount of unique sequence, as well as the total amounts of the genome sequence accounted for at each repeat copy-number, vary among the five strains indicating that the strains differ in their repeat structure and numbers of multicopy elements (Table 1). The choice of *k* limits the shortest repeat that can be resolved. Our choice of *k* = 21 allows resolution of the small repeated elements that typically occur in *E. coli* and other bacteria. Resolution of the repeat structure of a genome also provides an estimate of the total genome size (Table 1, bottom row). Despite different values of *k* providing different interpretations of repeat structure (Additional file 1: Table S1), estimates of genomes size are almost unaffected. Other choices for *k* from 15 to 29 changed the genome size estimates among our five *E. coli* datasets by less than 0.25% (Additional file 2: Table S2).

A common characteristic of the repeat structures of these *E. coli* genomes is a trend towards less unique sequence at higher copy numbers, except around seven times the abundance of the principal peak. The peaks at this copy number correspond to the *rrn* operons and any other sequence repeated a similar number of times. The *rrn* operons are present in seven copies in virtually all strains of *E. coli* and were observed in the 21-mers counted seven times in the completely sequenced *E. coli* DH1 genome (Table 1).

Despite the similarities in repeat structure, 21-mer abundance analysis using Kmerspectrumanalyzer allows differences in repeat structure between these five novel *E. coli* genomes to be resolved. The amount of unique sequence at six to eight copies per genome ranges from 4.8 kb in strain A_03_34 to 16.1 kb in strain E_01_37. While *E. coli rrn* operons are approximately 5.4 kb and expected to be present at seven copies, they are often non-identical at a few positions. Because 21-mer abundance analysis only resolves identical sequence repeats, the two smallest estimates of sequence at six to eight copies were 4.8 kb in strain A_03_34 and 4.9 kb in strain B_04_28 may not be an underestimation but an accurate reflection of the identical sequence among *rrn* operons. Any differences among the *rrn* operon sequences would be included in the total genome size estimate but at copy numbers less than seven. The estimates of sequence at six to eight copies in strains C_04_22, D_04_22 and E_01_37 were 9.1, 8.9 and 16.1 kb respectively and are greater than can be explained by the presence of seven *rrn* operons alone. These larger estimates are evidence of additional repeated sequences at abundances similar to that of the *rrn* operons.

There is little sequence repeated at high copy numbers within strains A_03_34 and B_04_28: the repeats in nine or more copies are represented by less than 121 bp of unique sequence in both cases. In contrast, strains D_04_22 and E_01_37 possess 1.3 kb and 3.0 kb respectively, of sequence repeated nine or more times. The 1.3 kb of high copy-number sequence from strain D_04_22 contains two protein-coding regions sharing high similarity with sequences annotated as IS911 transposases (GenBank accession number AY555729.1), and some of the 3-kb sequence at high copy number in strain E_01_37 shares high sequence similarity to plasmid pCE10B from *E. coli* O7:K1 strain CE10 (GenBank accession number: CP003036.1).

### Accuracy of 21-mer analysis for determining microbial genome sizes

The accuracy of estimates of total genome size obtained by our fitting procedure was tested in three ways. First, we assessed the sensitivity for discriminating the genome sizes from the whole-genome shotgun short-read datasets of five novel strains of *E. coli*, whose genome sizes were also estimated by totaling the lengths of I-CeuI restriction fragments resolved by pulsed-field gel electrophoresis (PFGE). Next, we tested the range of microbial genome sizes over which the procedure remains accurate by comparing the size estimates made from publicly available short-read datasets to the lengths of the corresponding complete genome sequences. Finally, we assessed the robustness of these genome size estimates by varying the read depths on target genomes and varying error content by quality-score-based read trimming.

The genome sizes obtained by enumerating 21-mers were very similar to those estimated by physically sizing I-CeuI restriction fragments by PFGE (Table 2). Based on PFGE, the strains ranged in size from 4.87 Mb (Strain A_03_34) to 5.50 Mb (Strain E_01_37), well within the size range of completely sequenced *E. coli* genomes (4.59 Mb to 5.86 Mb). On average, genome sizes obtained by PFGE differed by only about 2.5% from those obtained by 21-mer analysis, with the largest disparity observed in *E. coli* strain D, for which we obtained genome size estimates of 5.22 Mb by 21-mer analysis and 5.42 Mb by PFGE. Aside from this strain, the two methods are in agreement with respect to the relative genome size estimates, identifying Strain A_03_34 as the smallest with increasing sizes through Strain B_04_28, Strain C_04_22, Strain D_04_27 and Strain E_01_37.

**Table 2:** **Total genome sizes of five *E. coli *strains estimated by PFGE and icosihenamer analysis**

	**Genome size (Mb)**				
**Method**	**Strain A_03_34**	**Strain B_04_28**	**Strain C_04_22**	**Strain D_04_27**	**Strain E_01_37**
PFGE^a^	4.869	5.047	5.149	5.423	5.268
21-mer analysis of sequencing reads	4.776	4.935	5.181	5.219	5.502

^a^Restriction digests of genomic DNAs with I-*Ceu*I endonuclease yielded seven restriction fragments for each genome, the sizes of which are listed in Additional file 3: Table S3.

To assess the accuracy of the PFGE estimates, we also sized *E. coli* strain MG1655, for which the genome sequence is available [34], by PFGE, and compared fragment lengths to those expected based on the I-CeuI sites in the sequenced genome. Except for the largest I-CeuI restriction fragment, fragment length sizes estimated by PFGE were very close to those predicted by *in silico* digestion of the genome and are listed in Additional file 3: Table S3 along with each fragment size for the novel *E. coli* strains. Due to the vagaries of resolving and sizing fragments > 1 Mb by PFGE, we estimated the size of the largest I-CeuI in the *E. coli* MG1655 genome to be 2.793 Mb, whereas it is actually 2.498 Mb according to published sequence [34].

Although the genome sizes determined by PFGE were similar to those estimated by 21-mer analysis of the short-read data, there remains some discrepancy between the two methods. One source of the difference is that the PFGE only accurately assesses the sizes of linear DNA fragments. Bacterial genomes often contain small circular plasmids that remain intact after I-CeuI digestion and these circular elements are not included when tabulating genome size. The reported lengths of complete genome sequences may differ from size estimates by 21-mer analysis of read-sets because sequences, such as those encoded on multicopy plasmids, are represented in read data in proportion to their actual molecular quantities. In contrast, published genome sizes do not accommodate such differences in copy number leading to disparities in the canonical genome size and actual DNA content of the cell.

**Figure 2:**
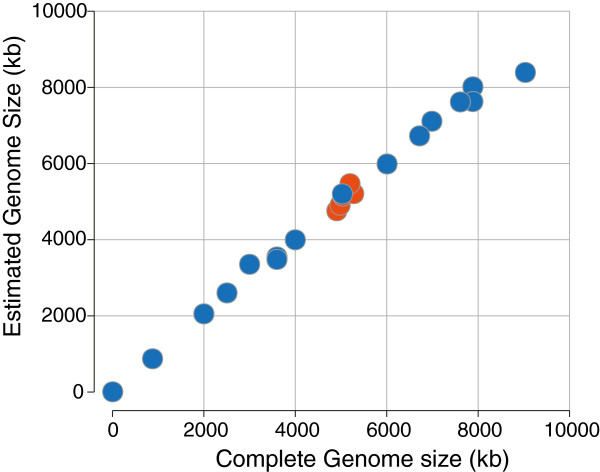
**Accuracy of genome sizes inferred from icosihenamer (21-mers) abundances.** Blue circles indicate genome sizes based on 21-mer abundance analysis of short-read datasets compared to published lengths of the same genomes. Red circles indicate genome sizes based on 21-mer abundance analysis compared to genome sizes for the identical strains estimated by PFGE. (See Additional file 4: Table S4 for list of genomes used in this analysis).

To further test the accuracy of *k-*mer analysis for estimating genome sizes, we applied the 21-mer abundance analysis to short-read datasets for a set of 19 microbial genomes whose known sizes range from 5,386 to 9,033,684 bp (Additional file 4: Table S4). The correlation coefficient between the actual sizes of these genomes and those estimated made by 21-mer abundance analysis is 0.997, with a root-mean-squared error relative to the known genome size of 6% (Figure 2). The largest absolute discrepancy of 642 kb was observed for *Niastella koreensis* GR20-10 (7% of the actual size) and the largest relative discrepancy of 11.7% was observed for *Listeria monocytogenes* J0161 (353 kb). Given the difference in the size of the *Niastella koreensis* (9 Mb), and *Listeria monocytogenes* (3 Mb) genomes, there is little relationship between the estimation error and the size of the genome over the range tested. However, any underlying relationship between the estimation accuracy and genome size may be masked by variation in systematic sequencing error among the datasets tested.

Some of the differences, particularly the overestimations of genome size by 21-mer analysis, may be caused by the presence of multicopy plasmids: these elements are always counted only once when reporting a completed genome size but are included in proportion to their actual copy numbers in 21-mer analysis. The over-estimate of the 7.6 Mb *Cylindrospermum stagnale* PCC 7417 genome (Additional file 4: Table S4) by only 6,600 bp is consistent with single copy plasmids and a small degree of error, and the genome size estimated for *Nostoc* sp. PCC 7524 could accommodate two copies of its smallest plasmid (6,361 bp) per chromosome. Similarly, the genome size estimated for *Thermovirga lienii* DSM 17291 accommodates two or three copies of its 31,872 bp plasmid in addition to its chromosome. *E. coli* KO11FL carries a single plasmid, pRK2, which has been shown experimentally to be maintained in *E. coli* strains at between 25 to 40 molecules per chromosomal equivalent [35,36]. We estimate the genome size of *E. coli* KO11FL to be 181,588 bp greater than its published chromosome size (5,021,812 bp), yielding ≈ 34 copies of the 5,360 bp pRK2 plasmid, in agreement with its known copy numbers. Figure 2 also displays the genome size estimates of the five novel *E. coli* strains as sized by PFGE demonstrating close agreement between the assessment of accuracy within a single species and across genome sizes of three orders of magnitude.

Our method is most accurate when read coverage over the entire genome is relatively even, since it provides *k*-mer abundance spectra with mixed Poisson distributions. One limitation in our approach is how well the proposed model can account for systematic errors in contemporary sequencing methods and still retain accuracy. Uneven read coverage broadens the peaks in the *k*-mer abundance spectra, sometimes causing errors in the assignment of sequences to a particular read coverage. Other factors that bias the *k*-mer abundance spectra include random sequencing errors and sequences derived from non-target DNA. Sequencing errors replace high-abundance sequences among reads with comparatively rare sequences creating many novel low-abundance *k*-mers. The fitting procedure implemented in Kmerspectrumanalyzer is designed to exclude these low-abundance *k*-mers by applying a heuristic low-coverage cutoff. We tested whether the genome sizes of the five novel *E. coli*, as estimated by 21-mer abundance analysis, were affected by removal of read positions likely to contain errors. We applied a trimming algorithm that retained the longest contiguous read positions with a less than 10% likelihood of being incorrect (i.e., a quality score of 10). This trimming procedure had little effect on the estimates of overall genome size, which changed by a maximum of only 0.19% (Additional file 5: Table S5). Therefore, Kmerspectrumanalyzer is sufficiently robust against sequencing error to negate any need for the pre-processing of read data for error mitigation.

The 21-mer abundance analysis described here would also be appropriate to the whole-genome shotgun sequencing datasets of eukaryotes of known ploidy. Although a *k*mer length of 21 is sufficient to ensure sparse sampling for microbial genomes, greater values for *k* may be necessary for the largest eukaryotic genomes. However, genomes with more complex repeat structure and lower sequencing coverage depths, can present challenges to the estimation approach presented here..In principle, this 21-mer abundance analysis can be applied to any sequencing methodology; however, there are some additional limitations. Extremely low coverage datasets (less than ≈ 10×) and datasets with very high error rates (>5%) contain insufficient sampling of the true *k*-mers to support numerical inferences. While numerical inferences are supported above 10× coverage depth, accuracy is compromised below about 75× coverage depth (Additional file 6: Figure S1). Among the four example datasets we tested, only the estimates for the *Owenweeksia hongkongensis* DSM 17368 reads (SRA run number: SRR190843) were relatively inaccurate between 75–400× coverage; however, it never differed by more than 4% from the completely assembled genome length. *k*-mer spectrum approaches can measure sequencing error, characterize heterozygosity, strain variation or mixtures of organisms [37] within a sample in an annotation-independent, scalable way, and therefore, are applicable to a several sequencing applications, including the interpretation of metagenomic data sets and diagnosing technical aspects of the sequencing procedure.

## Conclusions

Sequence repeats severely hamper the assembly of most genomes, and these repeats continue to obscure genome structure even with the high read depths afforded by new sequencing technologies. We provide a simple and rapid means to resolve the repeat structure and total size of a genome by directly analyzing the abundance of distinct *k*-mers among short reads. By obtaining the genome size and repeat structure of environmental isolates of *E. coli* from 76-bp *Illumina* reads, we demonstrate that the sensitivity of this method is great enough to resolve differences among bacterial strains. Total genome size estimates corresponded well with those obtained for the same strains by long-range RFLP mapping on pulsed-field gels. In addition, inferences of genome size from short-read datasets are not limited to strains within species but are accurate across a wide range of genome sizes from 0.005 to 9 Mb. The fitting procedure introduced here depends only on the *k*-mer spectrum, which summarizes the sequence redundancy of the data set but preserves none of the sequence content.

Previous work on plants [38] and humans [39] has exploited *k*-mer indices of raw sequence data to make inferences about the genome structure, particularly about the frequencies and contents of repetitive elements. More current analyses based on high-throughput sequencing data have proposed a variety of heuristics for estimating genome size by identifying features in the *k*-mer spectrum [21] or by using arbitrary cutoffs in the *k*-mer spectrum [40]. However, such approaches have failed to exploit the fact that repetitive sequences must occur at integer multiples of single copy sequences. By incorporating this information, our method implements a more biologically accurate model of the *k*-mer abundance distribution, adapting the procedure of [22] to high-coverage (>30×), short-read sequences.

Accurate estimates of total unique sequence from raw reads, as provided by Kmerspectrumanalyzer, can also aid in the optimization of read assemblies. Whereas the accuracy of an assembly is generally more important than its assembled length, the extent to which the total length of assembled contigs accounts for the actual length of the sequenced genome is a potential metric for the completeness of an assembly. The length of the target sequence is usually unknown, but it can be obtained directly from raw reads by 21-mer analysis, thereby allowing an absolute measure of assembly success. Moreover, abundance distributions in *k*-mer spectra are also useful in identifying samples that are dominated by PCR artifacts, samples contaminated with sequencing adapters, and samples that contain mostly positive-control calibration genomes (*e.g*., PhiX174, in the case of *Illumina*) without performing assembly or similarity searches.

We have observed that the reliability of many widely used statistics for summarizing the *k*-mer spectrum, including the number of unique *k-*mers, the fraction of *k-*mers that are unique, or the fraction of *k-*mers above an arbitrary or model-fitted threshold, are sensitive to changes in sampling depth. Two summary statistics that appear to be stable descriptions of certain informative properties of the *k*-mer spectrum are (*i*) the rank order distribution of *k*-mers and (*ii*) the cumulative fraction of the data consumed by rank ordered *k*-mers. In addition to genome repeat structure resolution, the Kmerspectrumanalyzer package includes tools to visualize the rank-order distribution of *k-*mers and assist in understanding sequence datasets, even in cases where fitting the spectrum to a mixed over-dispersed Poisson model fails.

Whole-genome shotgun sequencing applies technologies that generate hundreds of millions of short reads and has recently become the most widely used tool in genome analysis [41]. We introduce a straightforward methodology that provides information about the repeat structure of genomes that is ordinarily missing from assemblies of short reads. This additional information offers new insights about genome diversity and evolution that can be gained through the analysis of novel datasets or through the re-analysis of the large volumes of archived short read.

## Methods

### *k*-mer counting in sequence reads

The abundances of all overlapping 21-bp sequences present in a set of whole-genome shotgun short-read sequences were counted using Jellyfish[32]*k*-mer counting library (*vers.*1.1.5). No read trimming or error-correcting algorithms were applied. The frequencies of different *k*-mers (in this case 21-mers) at each abundance value contained in a set of sequences are plotted as a *k*-mer abundance spectrum (sometimes referred to as an abundance or coverage histogram).

A repeated sequence in a sampled genome affects the shape of these *k*-mer abundance spectra depending on its length and copy number. A sequence of length *l* will contain (*l – k +*1) different *k-*mers if it does not contain repeats of length greater than *k* – 1. If the same sequence occurs *n* times in a genome, shotgun sequencing would sample these *k*-mers *n* times more often than those that are single-copy in a genome, resulting in (*l – k +*1) *k*-mers with abundances *n* times higher than the average read-depth based on the number of sequencing reads. Therefore, repeated sequences in the genome results in higher abundances of the corresponding *k*-mers. These collections of *k*-mers at higher-than-normal abundances appear as multiple peaks at different positions along the *x*-axis of the *k*-mer abundance spectrum.

### Relating *k*-mer abundance to genome size and repeat abundance

We modeled the abundance distribution of *k-*mers as a mixture of over-dispersed Poisson (negative binomial) distributions in which the mixture coefficients represent amounts of sequence at each copy number and the overdispersion parameter accommodates uneven read-depth across the target genome. We refer to the peak in the abundance spectrum that consists of the *k*-mers corresponding to single-copy sequence in the target genome as the principal peak. Peaks in the abundance spectrum that have greater abundance than the principal peak correspond to sequences with different levels of abundance (i.e., repeat copy numbers), which are expected to occur at integer multiples of the principal abundance.

If *k*-mers were randomly sampled from a genome without repeats, the shape of the *k*-mer abundance spectrum would be a single Poisson distribution [22]:

$$P_{1}\left(x;a_{1,}c\right)=a_{1}\text{Poisson}\left(x;c\right),$$

where *P*_1_ (*x*) represents the number of *k*-mers observed *x* times, *a*_1_ is the number of unique *k-*mers, and *c* is a parameter describing the abundance of the principal peak. This model is expanded to include a mixture of components {*a*_1_, *a*_2_, … *a*_*n*_}, denumerated by *n,* that describe the number of sequences at each integer level of abundance by summing Poisson distributions for each abundance level:

$$P_{N}\left(x;c,\left\{a_{n}\right\}\right)=\sum_{n}a_{n}\;\text{Poisson}\left(x;c^{*}n\right),$$

which terminates at the number of occurrences of the highest-abundance *k*-mer in a genome (or at some prescribed cutoff). We interpret the mixing coefficients {*a*_1_, *a*_2_, … *a*_N_} as estimates of the amount of unique sequence at each repeat copy number. Finally, this can be generalized to over-dispersed Poisson shapes by introducing a single over-dispersion shape parameter *s* to allow distributions with excess variance:

$$\begin{array}{c} P_{\text{NO}}\left(x;c,\left\{a_{n}\right\},s\right)=\sum_{n}a_{n}\;\text{NegBinomial}\\ \;\;\;\left(x;\text{mean}=c*n;\text{alpha}=s/n\right). \end{array}$$

*NegBinomial* is the mu-alpha parameterization of the negative binomial, where *NegBinomial* (*x*; mu, alpha) gives the negative binomial distribution with mean = mu and variance = (1 + alpha)*mu^2^. When comparing plots of the actual 21-mer spectrum of *E. coli* K12 to the *P*_*N*_ and *P*_*NO*_ models calculated from 21-mer counts in the reference genome, we found that the mixed-Poisson model showed multiple peaks that fail to match the shape and general character of the peaks in the actual spectrum, whereas the over-dispersed adequately models the shape of the peaks of the spectrum.

We use this mixed Poisson with over-dispersion model to infer the *k*-mer-abundance distribution by maximizing the likelihood. Thus, for an observed *k*-mer spectrum *z*_*i*_:

$$\text{Likelihood}\left(c,\left\{a_{n}\right\},s|z_{i}\right)=\sum_{i}\;\text{Poisson}\left(z_{i};P_{\text{NO}}\left(i;\text{cov},\left\{a_{n}\right\},s\right)\right).$$

Here, the sum includes the full range of observed *k*-mer abundances, including values of *i*, for which *z*_*i*_ is zero, and *P*_*NO*_ ( ) is the sum of terms defined above.

After the set of coefficients {*a*_*n*_} has been estimated, the number of unique *k-*mers is the sum of the *a* coefficients:

$$N_{\text{unique}}=\sum a_{n}.$$

The estimate of the genome size in base pairs is the sum of the products of the unique *k*-mers and their relative abundances. This estimate is obtained by weighting *k*-mers by the occurrence number *n* before adding them up *i.e.*, the amount of unique sequence at each copy number multiplied by the copy-number:

$$G_{\text{sizeest}}=\sum n\;a_{n}.$$

### Fitting and implementation

The first three terms in the equations above, *a*_*1*_, *c*, and *s*, describe the height, abundance, and width of the principal peak in the *k*-mer spectrum. Twenty-nine additional terms, fitted sequentially, describe the height of peaks centered at integer multiples of the abundance of the principal peak ranging from 2× to 30×. Although sequencing errors are not explicitly modeled, by excluding points in the abundance spectrum at abundances less than half of the fitted abundance of the main peak, low-abundance sequencing artifacts effectively do not affect model fitting. We tested this assertion by estimating genome sizes with and without the quality score-based trimming of read positions. *DynamicTrim* from the **SolexaQA** package version 2.2 [42] was used to trim reads. The fitting procedure also employs several heuristics, including the masking of parts of the spectrum that are out of the range of abundances being fitted, weighted-least-squares optimization to set initial values, non-negativity constraints on the sequence size parameters, and successive fitting of low-order (and lower-dimensional) models before high-order models, all of which serve to stabilize the maximum likelihood optimization on real data.

Short-read datasets were downloaded from the NCBI Sequence Read Archive [43] and the corresponding complete genome sequences were obtained from the NCBI RefSeq database [44]. For this analysis, the smallest genome size was that of phiX174 (5,386 bp) and the largest was *Niastella koreensis* GR20-10 (9,033,684 bp). To fit the phiX174 genome, the procedure required a manual low-abundance cutoff of 10,000× and was constrained to fit only 1 term in the mixture model because of the extremely high coverage in that dataset (100,000×); otherwise, all of the microbial genomes were analyzed with the same procedures and parameters. To aid in the interpretation of the *k*-mer spectra (and the estimation of repeat copy numbers) for the five *E. coli* strains, we also queried and tabulated the amounts of repeated sequence in the complete genome sequence of *E. coli* DH1 (NC_017625).

The fitting procedure was implemented in Python 2.7.2 using Numpy, Scipy, and Matplotlib. An open-source implementation of this tool, Kmerspectrumanalyzer, and the scripts used to retrieve, process, and produce the numerical data in these analyses are available at http://github.com/MG-RAST/kmerspectrumanalyzer.git[45]. The tool is available as a module in the KBase sequence analysis framework [46], allowing end-users to perform these analyses on a third-party, scalable computing infrastructure rather than their own hardware. Motivated by previous work [47] our procedure is intended to be conveniently reproducible.

### Sampling, isolation and strain characterization of environmental *Escherichia coli*

Strains of *Escherichia coli* were isolated from liquid samples collected in July 2008 at the Central Contra Costa Sanitary District Treatment Plant in Martinez, California. Samples were titrated to a final concentration of 15% glycerol and stored at −80°C. An aliquot of each sample was diluted 1:100 in LB broth, and 100 μl was plated on MacConkey agar and incubated overnight at 37°C.

Colonies of *E. coli* were initially selected based on colony morphology and then typed genetically by sequencing fragments of three diagnostic loci, *fumC, gyrB* and *adk*, used in the multilocus sequence typing (MLST) analysis of *E. coli*[48]. MLST proceeded by the colony PCR [49] using primer pairs: *fumC* forward 5′-TCA CAG GTC GCC AGC GCT TC-3′ and *fumC* reverse 5′-GTA CGC AGC GAA AAA GAT TC-3′; *gyrB* forward 5′-TCG GCG ACA CGG ATG ACG GC-3′ and *gyrB* reverse 5′-ATC AGG CCT TCA CGC GCA TC-3′, *adk* forward 5′-ATT CTG CTT GGC GCT CCG GG-3′ and *adk* reverse 5′-CCG TCA ACT TTC GCG TAT TT-3′. PCR fragments were verified by agarose gel electrophoresis, and prepared for sequencing through the addition of 0.2 μl exonuclease and 0.2 μl calf intestinal phosphatase. Sanger sequencing of purified PCR products was performed at the University of Arizona Genetics Core for Sanger sequencing. Strains that differed in nucleotide sequence from any of the >3000 MLST-typed *E. coli* at any of the three diagnostic loci were deemed as “*unique*” and stored in LB broth supplemented with 15% glycerol and stored at −80°C.

### Whole-genome shotgun sequencing

Unique strains were streaked onto MacConkey agar and grown overnight at 37°C. Individual colonies were transferred to 2 ml LB broth and grown overnight at 37°C. DNA was isolated by disrupting pelleted cells in 1 ml of TES buffer containing 50mM NaCl, 50 mM Tris–HCl, 50 mM EDTA, 5% SDS (pH 7.6) followed by mechanical lysis with 0.1 mm zirconia/silica beads (BioSpec). The aqueous phase was removed, and treated with an equal volume of phenol/chloroform/isoamyl alcohol pH 7.9 (Ambion), followed by chloroform extraction and the isopropanol precipitation of DNA. Purified DNAs were quantified with Quant-iT PicoGreen (Invitrogen) and submitted to the Yale Center for Genome Analysis for library preparation and sequencing. Whole-genome shotgun sequencing was performed on an Illumina HiSeq 2000 generating 76-bp paired reads from the ends of 155-bp fragments. Short-read data were processed with the CASAVA 1.8.2 package.

### Sizing genomes by pulsed-field gel electrophoresis

Estimates of the genome sizes of the five newly sequenced strains of *E. coli* and of a control strain (*E. coli* MG1655) were determined by pulsed-field gel electrophoresis following methods described in [50]. In short, cells were grown in 5 ml of LB broth and treated with 180 μg/ml chloramphenicol to align chromosomes. Cells were harvested by centrifugation, washed and resuspended in 0.5 ml of TEN (10 mM Tris [pH 7.5], 100 mM EDTA [pH 8], 250 mM NaCl), and mixed with 0.75 ml of 1.5% low melt temperature agarose (RPI, Mount Prospect, IL) in TEN. Agarose plugs were incubated for 21 hours in lysis solution (0.1% lysozyme, 0.002% RNase, 0.5% Sarkosyl, 10 mM Tris [pH 7.5], 100 mM EDTA [pH 8], 250 mM NaCl), with subsequent overnight incubation at 45°C in 250 mM EDTA containing 0.1% proteinase K and 1% Sarkosyl. To inactivate excess proteases, agarose plugs were incubated in 1 mM PMSF for 1 hr, and washed and stored in 10mM Tris, 100 mM EDTA (pH 8) at 4°C. Agarose plugs were washed five times, each for 20 min, in 50 volumes of distilled H_2_O, and equilibrated in NEBuffer 4 with 0.1 mg/ml BSA (New England BioLabs, Ipswich, MA). Five units of I-CeuI restriction endonuclease (NEB) was added to initiate digestion, and after incubation overnight at 37°C, EDTA was added to each sample to a final concentration of 0.1 M to terminate digestion.

Electrophoresis was performed on a CHEF-DR II apparatus (Bio-Rad Laboratories, Richmond, CA) in 0.5× Tris-borate-EDTA at 14°C. To resolve DNA fragments in the 20 to 200 kb range, pulse durations were ramped from 5 sec to 12.5 sec; for DNA fragments in the 400 to 800 kb range, pulse durations were ramped from 60 sec to 100 sec. In both cases, electrophoresis proceeded for 24 hr in a 0.9% agarose gel at 6 V/cm. To resolve DNA fragments in the 2000 to 3000 kb range, pulse durations were ramped from 600 sec to 960 sec for 90 hr in a 0.7% agarose gel at 2.4 V/cm. Gels were stained and photographed digitally, and TIFF files of these images were loaded using the tifffile.py Python module (version 2013.01.18, [51]). Fragment sizes were estimated by interpolation to standards of known size using second order splines implemented in Scipy. Python 2.7.2 source code is included in the Kmerspectrumanalyzer repository in the ‘pfge_analysis’ folder [45].

## Abbreviations

IS: Insertion sequence; REP: Repetitive extragenic palindromic; ERIC: Enterobacterial repetitive intergenic consensus; BIME: Bacterial interspersed mosaic elements; Rrn: Ribosomal DNA.

## Competing interests

The authors declare that they have no competing interests.

## Authors’ contributions

FM, HO, WT, DW conceived of the study; HO collected samples; MS, DW characterized and prepared bacterial samples; WT, DW analyzed sequence data; WT implemented Kmerspectrumanalyzer; DW, HO and WT wrote the manuscript. All authors read and approved the final manuscript.

## Supplementary Material

Additional file 1: Table S1 Size and repeat structure of the *E. coli* DH1 genome sequence using *k*-mers of different size. Click here for file

Additional file 2: Table S2 Genome sizes estimated from read sets of *E. coli* strains using *k*-mers of different size. Click here for file

Additional file 3: Table S3 I-*Ceu*I fragment lengths for *E. coli* strains. Click here for file

Additional file 4: Table S4 Estimates of microbial genome sizes based on *k*-mer analysis of short read datasets. Click here for file

Additional file 5: Table S5 Effect of quality-score-based trimming on genome size estimates. Click here for file

Additional file 6: Figure S1 Ratio of assembled to estimated genome sizes at different read depths. Click here for file
