# Supplementary material for: Rapid quantification of sequence repeats to resolve the size, structure and contents of bacterial genomes
Source: BMC Genomics. 2013 Aug 8;14:537. doi: 10.1186/1471-2164-14-537 (PMC3751351; doi:10.1186/1471-2164-14-537)
Supplement: Additional file 1: Table S1 — Size and repeat structure of the E. coli DH1 genome sequence using k-mers of different size. [file 1471-2164-14-537-S1.doc]

**Table S1. Size and repeat structure of the *E. coli* DH1 genome sequence using *k*-mers of different size**

|  | **Total sequence (bp)** | | | | | | | |
| --- | --- | --- | --- | --- | --- | --- | --- | --- |
| **Copy number** | ***k* = 15** | ***k* = 17** | ***k* = 19** | ***k* = 21** | ***k* = 23** | ***k* = 25** | ***k* = 27** | ***k* = 29** |
| 1x | 4,343,067 | 4,472,277 | 4,489,279 | 4,494,886 | 4,498,585 | 4,501,634 | 4,504,264 | 4,506,621 |
| 2x | 162,178 | 45,060 | 31,810 | 29,196 | 28,098 | 27,300 | 26,674 | 26,144 |
| 3x | 28,908 | 21,969 | 21,351 | 20,877 | 20,412 | 19,989 | 19,611 | 19,248 |
| 4x | 10,836 | 9,164 | 8,620 | 8,288 | 7,892 | 7,512 | 7,124 | 6,784 |
| 5x | 10,080 | 9,770 | 9,585 | 9,370 | 9,250 | 9,130 | 9,080 | 9,025 |
| 6x | 8,286 | 8,268 | 8,418 | 8,490 | 8,508 | 8,616 | 8,664 | 8,754 |
| 7x | 37,016 | 36,918 | 36,449 | 35,924 | 35,427 | 34,944 | 34,475 | 33,999 |
| 8x | 3,368 | 2,408 | 1,944 | 1,704 | 1,456 | 1,240 | 1,056 | 904 |
| 9x | 297 | 189 | 234 | 207 | 198 | 153 | 135 | 126 |
| 10x | 390 | 330 | 350 | 260 | 200 | 180 | 170 | 150 |
| 11x – 20x | 20,469 | 19,820 | 19,152 | 18,757 | 18,535 | 18,372 | 18,224 | 18,078 |
| 21x – 70x | 5,798 | 4,518 | 3,497 | 2,728 | 2,124 | 1,613 | 1,204 | 846 |
| Total^a^ | 4,630,693 | 4,630,691 | 4,630,689 | 4,630,687 | 4,630,685 | 4,630,683 | 4,630,681 | 4,630,679 |

^a^ The size of the sequenced *E. coli* DH1 genome is 4,630,707 bp, *k* ­– 1 greater than the total *k*-mers.
