# Supplementary material for: Rapid quantification of sequence repeats to resolve the size, structure and contents of bacterial genomes
Source: BMC Genomics. 2013 Aug 8;14:537. doi: 10.1186/1471-2164-14-537 (PMC3751351; doi:10.1186/1471-2164-14-537)
Supplement: Additional file 2: Table S2 — Genome sizes estimated from read sets of E. coli strains using k-mers of different size. [file 1471-2164-14-537-S2.doc]

**Table S2. Genome sizes estimated from read sets of *E. coli* strains using *k*-mers of different size**

|  | **Estimated genome size (bp)** | | | | | | | |
| --- | --- | --- | --- | --- | --- | --- | --- | --- |
| ***E. coli* strain** | ***k* = 15** | ***k* = 17** | ***k* = 19** | ***k* = 21** | ***k* = 23** | ***k* = 25** | ***k* = 27** | ***k* = 29** |
| A_03_34 | 4,778,825 | 4,776,822 | 4,776,195 | 4,775,705 | 4,775,453 | 4,774,872 | 4,774,274 | 4,773,837 |
| B_04_28 | 4,927,099 | 4,932,857 | 4,934,298 | 4,935,111 | 4,933,859 | 4,935,175 | 4,934,608 | 4,936,041 |
| C_04_22 | 5,168,076 | 5,175,804 | 5,177,972 | 5,180,594 | 5,182,109 | 5,183,615 | 5,184,004 | 5,185,904 |
| D_04_27 | 5,216,262 | 5,218,504 | 5,219,054 | 5,219,387 | 5,219,365 | 5,219,058 | 5,216,893 | 5,216,055 |
| E_01_37 | 5,504,417 | 5,503,399 | 5,502,931 | 5,502,511 | 5,502,004 | 5,501,319 | 5,500,696 | 5,500,121 |
