# Supplementary material for: Rapid quantification of sequence repeats to resolve the size, structure and contents of bacterial genomes
Source: BMC Genomics. 2013 Aug 8;14:537. doi: 10.1186/1471-2164-14-537 (PMC3751351; doi:10.1186/1471-2164-14-537)
Supplement: Additional file 3: Table S3 — I-CeuI fragment lengths for E. coli strains. [file 1471-2164-14-537-S3.doc]

**Table S3. I-*Ceu*I fragment lengths for *E. coli* strains**

| **Fragment lengths (bp)**  **estimated by PFGE** | | | | | |  | **Fragment lengths (bp) based on genome sequence** |
| --- | --- | --- | --- | --- | --- | --- | --- |
| **Strain A_03_34** | **Strain B_04_28** | **Strain C_04_22** | **Strain D_04_27** | **Strain E_01_37** | ***E. coli* MG1655** |  | ***E. coli* MG1655** |
| 40,036 | 41,747 | 40,720 | 41,062 | 37,299 | 40,378 |  | 41,398 |
| 115,992 | 94,779 | 130,161 | 120,097 | 117,873 | 93,239 |  | 93,812 |
| 139,086 | 136,862 | 120,782 | 179,801 | 138,060 | 133,099 |  | 131,117 |
| 525,392 | 540,151 | 530,411 | 538,598 | 547,142 | 528,111 |  | 520,769 |
| 680,359 | 645,404 | 860,359 | 711,042 | 708,712 | 663,270 |  | 657,364 |
| 683,855 | 720,752 | 733,957 | 831,831 | 812,800 | 702,109 |  | 697,595 |
| 2,684,174 | 2,866,877 | 2,939,489 | 3,000,390 | 2,905,916 | 2,827,057 |  | 2,497,592 |
