# Supplementary material for: Rapid quantification of sequence repeats to resolve the size, structure and contents of bacterial genomes
Source: BMC Genomics. 2013 Aug 8;14:537. doi: 10.1186/1471-2164-14-537 (PMC3751351; doi:10.1186/1471-2164-14-537)
Supplement: Additional file 4: Table S4 — Estimates of microbial genome sizes based on k-mer analysis of short read datasets. [file 1471-2164-14-537-S4.doc]

**Table S4. Estimates of microbial genome sizes based on *k*-mer analysis of short read datasets**

| **NCBI SRA^a^**  **run number** | **Genome source** | **Genome size^b^ (kb)** | **Number of replicons** | **Genome size estimate (kb)** | **Coverage estimate** | **Unique estimate (kb)** |
| --- | --- | --- | --- | --- | --- | --- |
| SRR059788 SRR059789 | *Niastella koreensis* GR20-10, DSM 17620 | 9,033.7 | 1 | 8,392.0 | 53.4 | 8,287.2 |
| SRR072318  SRR090709 | *Burkholderia* sp. CCGE1002 | 7,884.9 | 4 | 8,014.3 | 24.3 | 7,676.8 |
| SRR031266  SRR031261  SRR031262  SRR031263  SRR031264  SRR031265  SRR031266 | *Burkholderia* sp. CCGE1002 | 7,884.9 | 4 | 7,627.3 | 104.6 | 7,410.5 |
| SRR610299 | *Cylindrospermum stagnale* PCC 7417 | 7,610.6 | 4 | 7,617.2 | 510.7 | 7,064.6 |
| SRR071425 | *Mycobacterium smegmatis* MC2 155 | 6,988.2 | 1 | 7,110.1 | 251.7 | 6,626.3 |
| SRR610309 | *Nostoc* sp. PCC 7524 | 6,718.9 | 3 | 6,726.8 | 177.2 | 6,162.0 |
| SRR090599 | *Planctomyces brasiliensis* IFAM 1448, DSM 5305 | 6,008.0 | 1 | 5,988.3 | 393.6 | 5,841.2 |
| SRR059232  SRR059233  SRR059234  SRR059235  SRR059236 | *Escherichia coli* KO11FL | 5,029.3 | 2 | 5,203.4 | 36.8 | 4,710.7 |
| SRR190843 | *Owenweeksia hongkongensis* DSM 17368 | 4,000.0 | 1 | 3,997.3 | 1,065 | 3,925.0 |
| SRR006332 | *Acinetobacter baylyi* ADP1 | 3,598.6 | 1 | 3,546.2 | 52.6 | 3,480.8 |
| SRR006330 | *Acinetobacter baylyi* ADP1 | 3,598.6 | 1 | 3,480.5 | 22.6 | 3,373.9 |
| SRR396647  SRR396649  SRR396650  SRR396651  SRR396653 | *Listeria monocytogenes* J0161, FSL R2-499 | 3,000.4 | 1 | 3,353.8 | 33.8 | 2,911.6 |
| SRR089543  SRR089544 | *Rothia dentocariosa* ATCC 17931 | 2,506.0 | 1 | 2,602.1 | 37.8 | 2,473.1 |
| SRR060959  SRR060960 | *Thermovirga lienii* DSM 17291 | 1,999.6 | 2 | 2,052.7 | 108.1 | 1,875.8 |
| SRR006331 | *Mycoplasma agalactiae* PG2 | 877.4 | 1 | 872.8 | 21.6 | 855.5 |
| SRR387449 | phiX174 | 5.4 | 1 | 5.3 | 171,200.0 | 5.284.0 |
| SRR769601 | *Escherichia coli* strain A_03_34^c^ | 4,908.0 |  | 4,759.9 | 54.6 | 4,642.5 |
| SRR769603 | *Escherichia coli* strain B_04_28^c^ | 4,980.0 |  | 4,908.5 | 68.5 | 4,821.3 |
| SRR769600 | *Escherichia coli* strain C_04_22^c^ | 5,039.0 |  | 5,145.4 | 80.3 | 4,585.4 |
| SRR769602 | *Escherichia coli* strain D_04_27^c^ | 5,278.0 |  | 5,209.8 | 60.5 | 5,004.6 |
| SRR769599 | *Escherichia coli* strain E_01_37^c^ | 5,196.0 |  | 5,445.7 | 84.5 | 4,801.5 |

^a^ NCBI SRA: National Center for Biotechnology Information Sequence Read Archive (http://www.ncbi.nlm.nih.gov/sra)

^b^ Genome size obtained by complete sequencing or by PFGE measurement.

^c^ Novel *E. coli* isolate, reported in this study.
