# Supplementary material for: Rapid quantification of sequence repeats to resolve the size, structure and contents of bacterial genomes
Source: BMC Genomics. 2013 Aug 8;14:537. doi: 10.1186/1471-2164-14-537 (PMC3751351; doi:10.1186/1471-2164-14-537)
Supplement: Additional file 5: Table S5 — Effect of quality-score-based trimming on genome size estimates. [file 1471-2164-14-537-S5.doc]

**Table S5. Effect of quality-score-based trimming on genome size estimates**

|  | **Combined length of reads (bp)** | |  | **Genome size estimate (bp)** | |  |
| --- | --- | --- | --- | --- | --- | --- |
| ***E. coli* strain** | **Raw reads** | **Trimmed reads^a^** | **Percentage**  **removed by**  **trimming** | **Raw reads** | **Trimmed reads^a^** | **Difference in genome size**  **estimates** |
| A_03_34 | 362,127,992 | 344,702,582 | 4.81 | 4,775,814 | 4,766,569 | -0.19% |
| B_04_28 | 468,434,968 | 446,724,279 | 4.63 | 4,935,386 | 4,927,683 | -0.16% |
| C_04_22 | 580,398,016 | 554,098,452 | 4.53 | 5,180,545 | 5,176,786 | -0.07% |
| D_04_27 | 437,355,832 | 417,108,122 | 4.63 | 5,219,319 | 5,210,466 | -0.17% |
| E_01_37 | 645,818,056 | 614,475,488 | 4.85 | 5,502,494 | 5,492,264 | -0.19% |

^a^ Sequence reads were trimmed with *Dynamic Trim* of the **SolexaQA** vers. 2.2 package. This procedure retains the longest contiguous region for which the probability of an incorrect base call remains below 10%. Estimates of incorrect base calls are based on the quality scores assigned to each read position during sequencing.
