# Supplementary material for: Rapid quantification of sequence repeats to resolve the size, structure and contents of bacterial genomes
Source: BMC Genomics. 2013 Aug 8;14:537. doi: 10.1186/1471-2164-14-537 (PMC3751351; doi:10.1186/1471-2164-14-537)
Supplement: Additional file 6: Figure S1 — Ratio of assembled to estimated genome sizes at different read depths. [file 1471-2164-14-537-S6.doc]

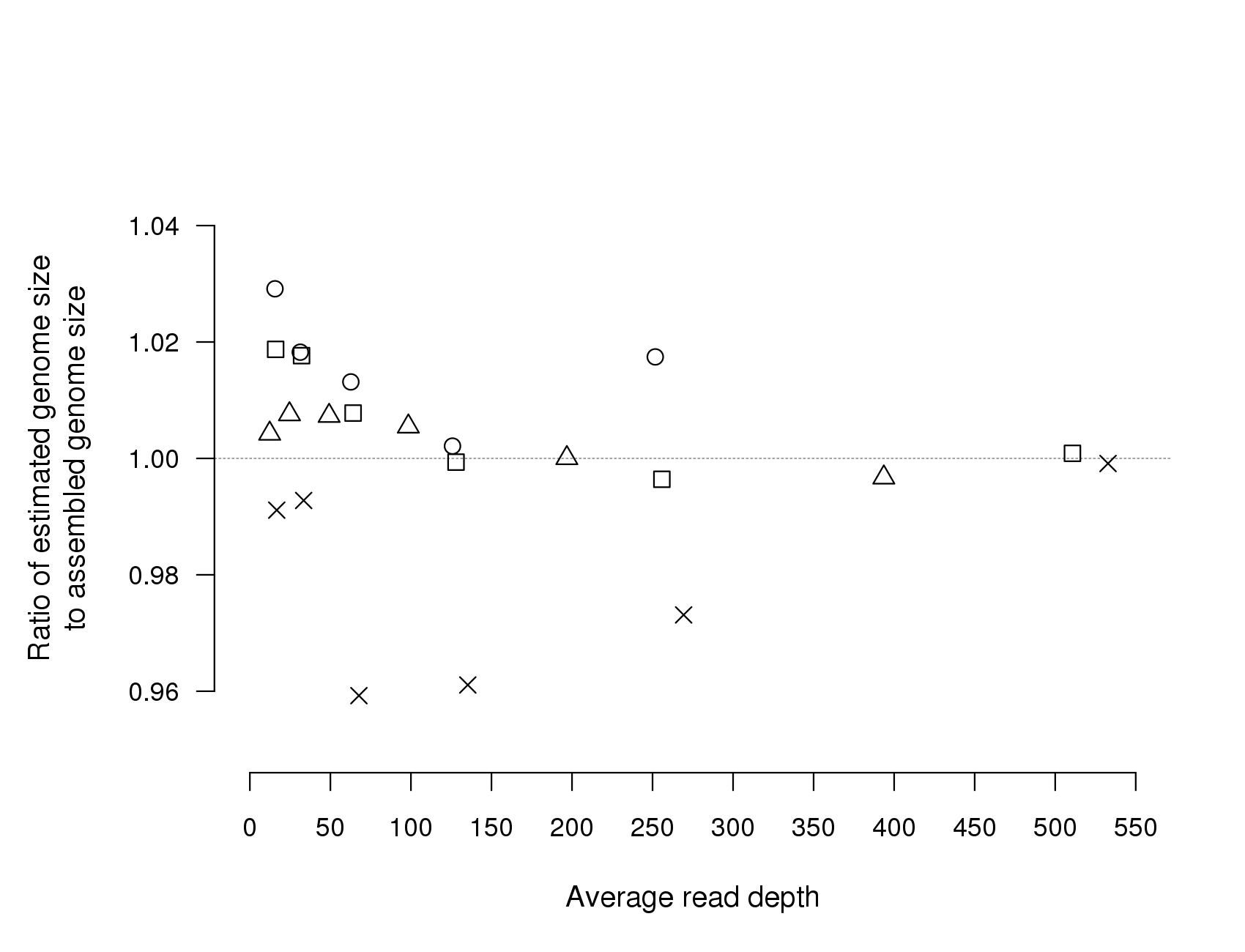
**Figure S1**. Ratio of assembled to estimated genome sizes at different read depths. Sample sets of different read depths were obtained by randomly selecting subsamples of short-read datasets at 0.5, 0.25, 0.125, 0.0625 and 0.03125 of their original sizes. Original dataset sizes were obtained from assembled sequences, and average read depths were estimated by 21-mer frequency analysis and correspond to the principle peak in a 21-mer spectrum. Symbols denote the organism from which the whole-genome shotgun sequence data were obtained: ○ *Cylindrospermum stagnale* PCC 7417 (SRA run number: SRR610299); △ *Mycobacterium smegmatis* MC2 155 (SRA run number: SRR071425); × *Owenweeksia hongkongensis* DSM 17368 [1] (SRA run number: SRR190843); □ *Planctomyces brasiliensis* IFAM 1448 DSM 5305 [2] (SRA run number: SRR090599).

**References**

1. Riedel T, Held B, Nolan M, Lucas S, Lapidus A, Tice H, Del Rio TG, Cheng JF, Han C, Tapia R, Goodwin LA, Pitluck S, Liolios K, Mavromatis K, Pagani I, Ivanova N, Mikhailova N, Pati A, Chen A, Palaniappan K, Rohde M, Tindall BJ, Detter JC, Göker M, Woyke T, Bristow J, Eisen JA, Markowitz V, Hugenholtz P, Klenk HP *et al.*: **Genome sequence of the orange-pigmented seawater bacterium Owenweeksia hongkongensis type strain (UST20020801(T))**. *Stand Genomic Sci* 2012, **7**:120-130.

2. Wu DY, Hugenholtz P, Mavromatis K, Pukall R, Dalin E, Ivanova NN, Kunin V, Goodwin L, Wu M, Tindall BJ, Hooper SD, Pati A, Lykidis A, Spring S, Anderson IJ, D'haeseleer P, Zemla A, Singer M, Lapidus A, Nolan M, Copeland A, Han C, Chen F, Cheng JF, Lucas S, Kerfeld C, Lang E, Gronow S, Chain P, Bruce D *et al.*: **A phylogeny-driven genomic encyclopaedia of Bacteria and Archaea.** Nature, 2009, **462**:1056-1060.
